# Research progress in the preparation of mesophase pitch from fluid catalytic cracking slurry

**DOI:** 10.1039/d3ra01726e

**Published:** 2023-06-20

**Authors:** Mingzhi Wang, Bei Yang, Tao Yu, Xiaoyan Yu, Muhammad Rizwan, Xulu Yuan, Xinyao Nie, Xiaolong Zhou

**Affiliations:** a International Joint Research Center of Green Energy Chemical Engineering, East China University of Science and Technology China; b Baowu Carbon Technology Co., Ltd. China; c Liaoning Qingyang Chemical Industry Corporation China

## Abstract

For the preparation of high-performance pitch-based carbon fibers and other carbon materials, mesophase pitch serves as a high-quality precursor. Since FCC (Fluid Catalytic Cracking) oil slurry is abundant in aromatic hydrocarbons and saturated hydrocarbons (about 95% in total), it has become an ideal choice for developing new carbon material products. This paper details the research progress of preparing mesophase asphalt with FCC oil slurry as a raw material from perspectives including the preparation method of synthesizing mesophase asphalt from FCC oil slurry, the impact factors of the formation process of mesophase asphalt and the industrial application of mesophase asphalt.

## Abstract 2

For the preparation of high-performance pitch-based carbon fibers and other carbon materials, mesophase pitch serves as a high-quality precursor.

## Graphical abstract

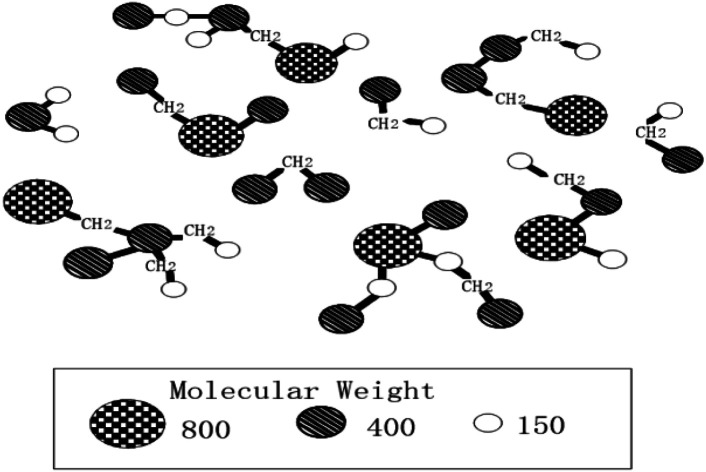

## 1. Introduction

New graphite materials, electrode materials, carbon materials and carbon fibers, in the modern chemical industry,^1–3^ have been widely used. With their high quality, availability and affordability,^4–6^ these materials have been reported across fields. Research into pitch-based carbon materials is centred on improving new production processes and preparing precursors with good performance, so that high-quality pitch-based carbon materials with low cost can be developed. As a precursor for the preparation of new carbon materials with excellent properties, mesophase pitch can be employed by improving the process conditions and adjusting the process methods to produce different carbon materials such as foamed carbon,^7–9^ carbon microspheres^10–12^ or needle coke.^13–15^ It is, notably, of great value for industrial applications. In addition to its abundant sources and affordable costs, the advantages of mesophase pitch also include easy graphitization, high oxidation activity, low softening point, and the ability to freely control the composition structure and melt viscosity;^16–19^ with these advantages, it is becoming increasingly utilized in industrial applications. At the current stage, the relative molecular weight of commonly used mesophase pitch ranges from 370 to 2000, which is about 3.5 times that of isotropic pitch, with a volatile content between 15% and 20%, a density from 1.4 g cm^−3^ to 1.6 g cm^−3^, and a hydrogen to carbon atom ratio between 0.35 and 0.5.^20^ In the preparation process of mesophase pitch, the top priority lies in the selection of raw materials. The main raw materials, at current stage, are coal pitch,^21–23^ aromatic compounds^24–26^ and petroleum pitch.^27–29^ Among them, the advanced process acts as the preparation of mesophase pitch from coal tar pitch, which is also of interest to researchers due to its high degree of condensation and reactivity. Coal pitch, however, contains a large number of heteroatoms, which will affect the formation of mesophase spheres during the preparation of mesophase pitch, and ultimately impact on the performance of the product;^30,31^ if aromatic compounds are used as raw materials to prepare mesophase pitch, the conditions of the preparation process can be quite demanding. Moreover, the aromatic hydrocarbon molecules themselves are difficult to react with, and the reaction cost and experimental difficulty greatly restrict its use in the synthesis of mesophase pitch.^32–34^ Petroleum asphalt, however, has attracted the attention of researchers^35,36^ due to its high content of aromatics, availability and affordability. Catalytic cracking slurry (FCC) is the product of the treatment and processing of crude oil, which contains residual solid residues and catalyst impurities. The utilization rate of its raw materials is therefore not that desirable, which greatly reduces the utilization value in practice.^37–39^ Nevertheless, FCC oil slurry contains a large amount of aromatic hydrocarbons and heavy components that can be used to prepare mesophase pitch and other high value-added chemical materials.^40–42^

## 2. Research status and advantages of mesophase pitch

### 2.1 Research status of mesophase pitch at home and abroad

At the beginning of the 21st century, domestic researchers developed high-quality carbon fiber products with an average tensile strength of 1200–1500 MPa and an average elastic modulus of 70–80 GPa using petroleum heavy oil as raw material and a set of mesophase pitch carbon fiber preparation technology. In recent years, the industrial production of 1.5 K continuous fibres with an average diameter of 11 μm, strength of 2400 MPa, modulus of 811 GPa and thermal conductivity of 600 W m^−1^ K^−1^ or more has been achieved in China.^43^ Foreign research on mesophase pitch carbon materials began in the 1960s. With the in-depth research and industrialization in this field, the core technology of mesophase pitch carbon materials is mainly controlled by Japanese graphite fiber, Japanese Mitsubishi Chemical and American Cytec. The mesophase pitch carbon fiber produced by the company has a maximum tensile strength of 3700 MPa, a maximum modulus of 966 GPa and a maximum thermal conductivity of 1170 W m^−1^ K^−1^. Shimanoe team^44^ put forward a new method for preparing mesophase pitch in 2020. The mesophase pitch is prepared by the process of three-step hydrogenation, heat treatment under nitrogen environment and thin layer evaporation. The prepared mesophase pitch carbon material boasts excellent mechanical properties, with strength up to 3 GPa and modulus up to 450 GPa.

### 2.2 Advantages of mesophase asphalt

The relative molecular weight of mesophase asphalt ranges from about 400 to 4000. The asphalt-like mixture composed of polycyclic aromatic hydrocarbons and heterocyclic aromatic hydrocarbons with a certain flatness and orderly arrangement is crystallographically optically anisotropic and in a fluid state. Because of its affordable cost, easy graphitization, wide sources and low softening point, mesophase pitch is common in industrial applications and can be used to prepare a variety of carbon materials. Professor Mochida of Japan once commented on the intermediate phase bitumen, which features the appropriate relative molecular mass and structure, high stability to recondensation and thermal decomposition reactions, and the ability to maintain fluidity, alignment and solubility over a long period of time. These advantages, admittedly, are quite conducive for the synthesis of carbon fibres. Several researchers found that mesophase pitch synthesized from FCC slurry is in the form of large melting body, with low softening point. There is also a large smooth flow region in its viscosity–temperature curve, with excellent spinnability.^20,28,33^

## 3. Formation mechanism of mesophase structure

**Fig. 1:**
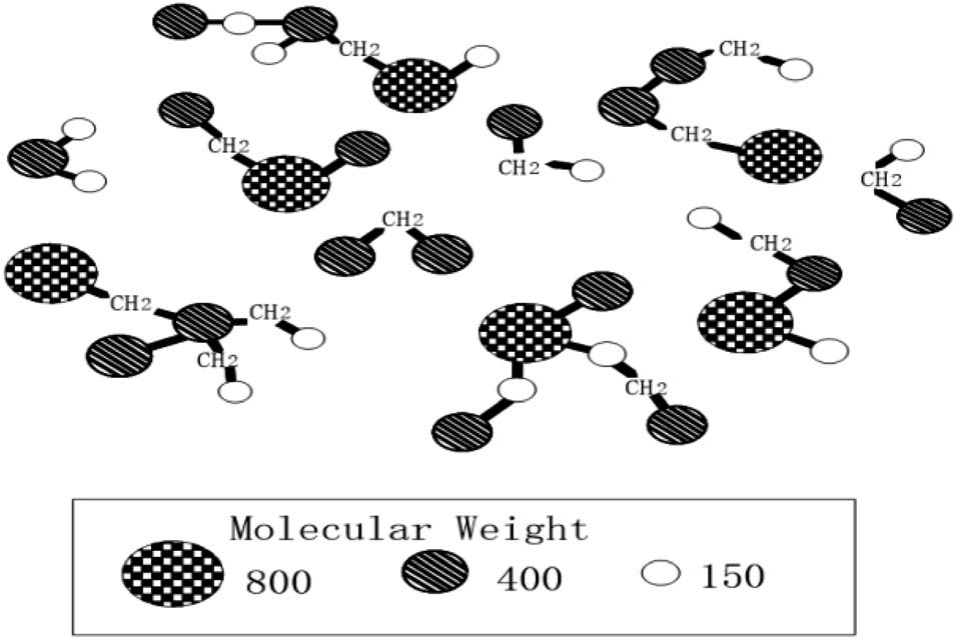
Cobweb structure model of mesophase.

**Fig. 2:**
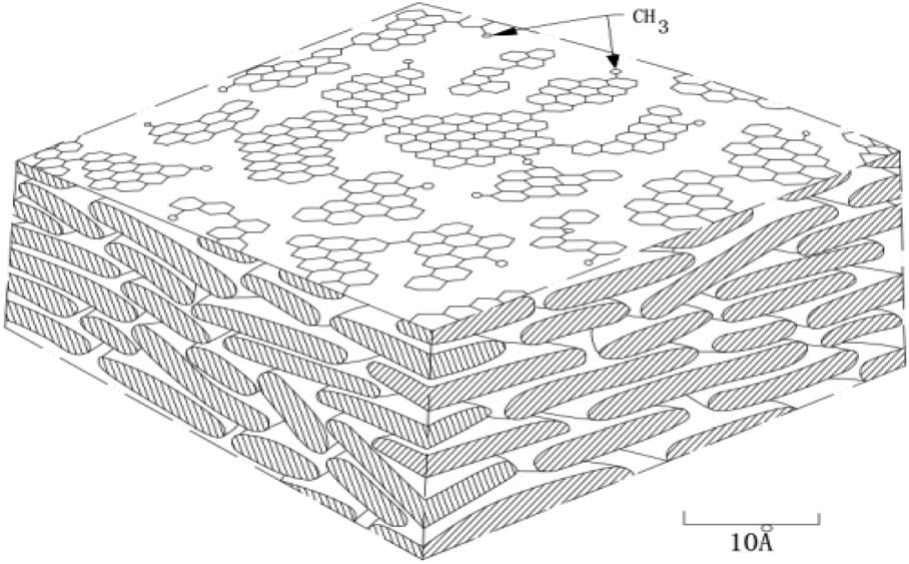
Three dimensional network structure model of mesophase.

In order to prepare carbon material precursors with better performance, quite a few researchers have conducted an in-depth research on the formation mechanism of mesophase pitch. In 1961, Taylor and other scholars^45^ discovered the regular motion of many mesophase particles with optical anisotropy when exploring coal coking. Subsequently, scholars from all over the world have been working on the study of mesophase mechanism including China's Wang Chengyang, Qian Shuan,^46^ the Lewis team^47–49^ in the United States and the Mochida research group^50^ in Japan, *etc.* Most of them, however, only inferred the reaction mechanism through the morphological characteristics of the samples before and after the experiment, and did not directly observe the morphological change of the reactants in each reaction stage. According to Chinese scholars' speculations, it is rather challenging to observe the specific states of the different stages because of the high heat treatment temperature and the rapid changes of the reactants' morphology. The formation of the intermediate phase is a result of the competition between two anisotropic reaction processes, namely, the competition between the reduced small molecules because of thermal decomposition and the formed planar fused-ring macromolecules due to polymerization of small molecules. Polymerization, in the formation of the mesophase, acts as a primary role.^51^ Early researchers speculated that the model of mesophase molecule was spider web structure model, as shown in Fig. 1; after further research, the researchers proposed a three-dimensional structure model of the mesophase, as shown in Fig. 2.

### 3.1 Traditional mechanism of mesophase formation

**Fig. 3:**
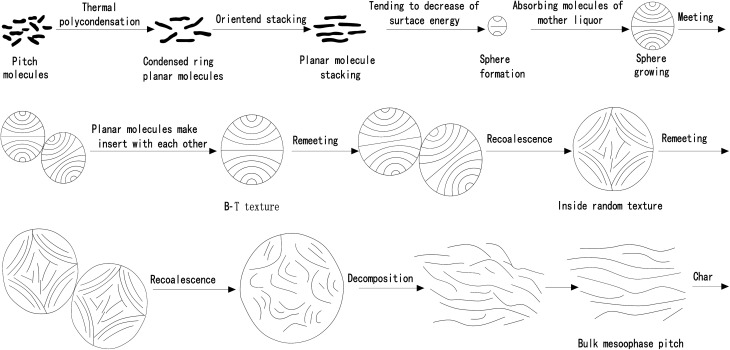
Traditional formation theory of mesophase asphalt.

Changes in the mesophase particles of pitch during heat treatment were observed with hot-stage polarized light microscopy. The raw material pitch is composed of various kinds of aromatic hydrocarbons.^52^ When the temperature is close to 400 °C, the raw material undergoes a series of reactions such as pyrolysis, dehydrogenation condensation, *etc.*, forming planar polynuclear aromatic hydrocarbon macromolecules. And the larger the molecular weight, the stronger the intermolecular force, the easier it is to associate with each other and aggregate into a stack. The stack then shrinks into small particles under the surface tension. The granular material absorbs the isotropic mother liquor and expands continuously, and under the action of tension, these granular materials are combined to generate larger granular materials. After the mesophase particles get combined and grown for several times, they gradually become larger and larger particles. When the particle size of the particles is larger than the critical value that the surface tension can maintain, the particles will decompose to form a mesophase. The whole process is presented in Fig. 3.

As a conventional theory, the theory of formation mechanism of mesophase remains flawed in a number of ways: (1) during the collision and growth of mesophase spheres, exceptionally high energy is required between molecular lamellae to insert each other, which is far too challenging to achieve. (2) The theory attributed the growth of mesophase spheres to the melting of spheres, which was not observed during the experiment. (3) The melting phenomenon of mesophase sphere is only applicable to the homogeneous nucleation system, while in the growth process of heterogeneous nucleation sphere, the melting phenomenon of sphere is not observed. The melting phenomenon only occurs when the sphere is deformed into a bulk mesophase.

### 3.2 The theory of “micro-domain construction”

**Fig. 4:**
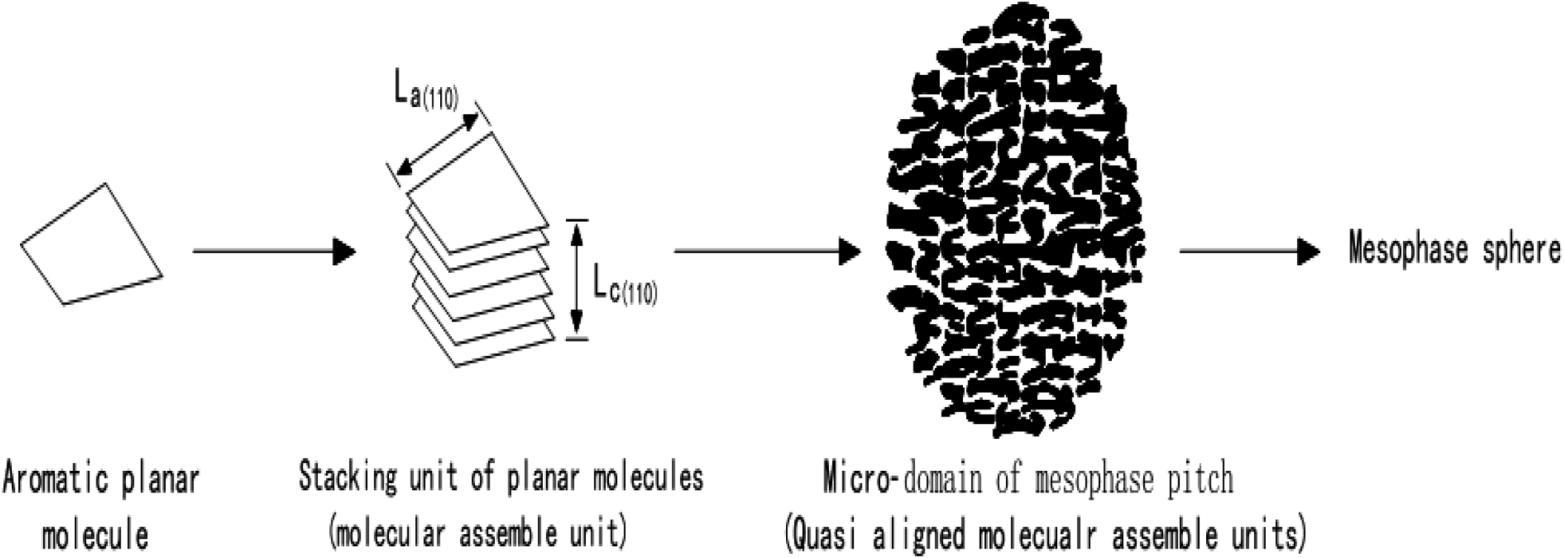
Explanation of the formation process of mesophase in the theory of “Micro Domain Construction”.

Mochida *et al.*^53^ put forward the “Micro-domain Construction Theory” when studying the axial microstructure in mesophase pitch-based graphite fibers, and made a new explanation for the growth and fusion process of pellets in the traditional mechanism. The theory points out that lamellar molecules composed of polycyclic aromatic hydrocarbons will gradually accumulate to form three-dimensional molecular assembly units, and then these accumulation units will continue to stack to form spherical domains, which will then stack and grow with each other, and finally form mesophase spheres. The process is as shown in Fig. 4.

“Domain Unit Construction Theory” not only fails to employ the unreasonable explanation that spheres are fused by the insertion of lamellar molecules, but also explains the growth process of mesophase spheres in homogeneous and heterogeneous nucleation systems respectively. The limitation, however, is that the introduced lamellar aromatic molecular stacking unit is only the average crystallite obtained by theoretical calculation, which is only an assumption and has not been observed in subsequent experiments.

### 3.3 Theory of “particle basic unit construction”

**Fig. 5:**
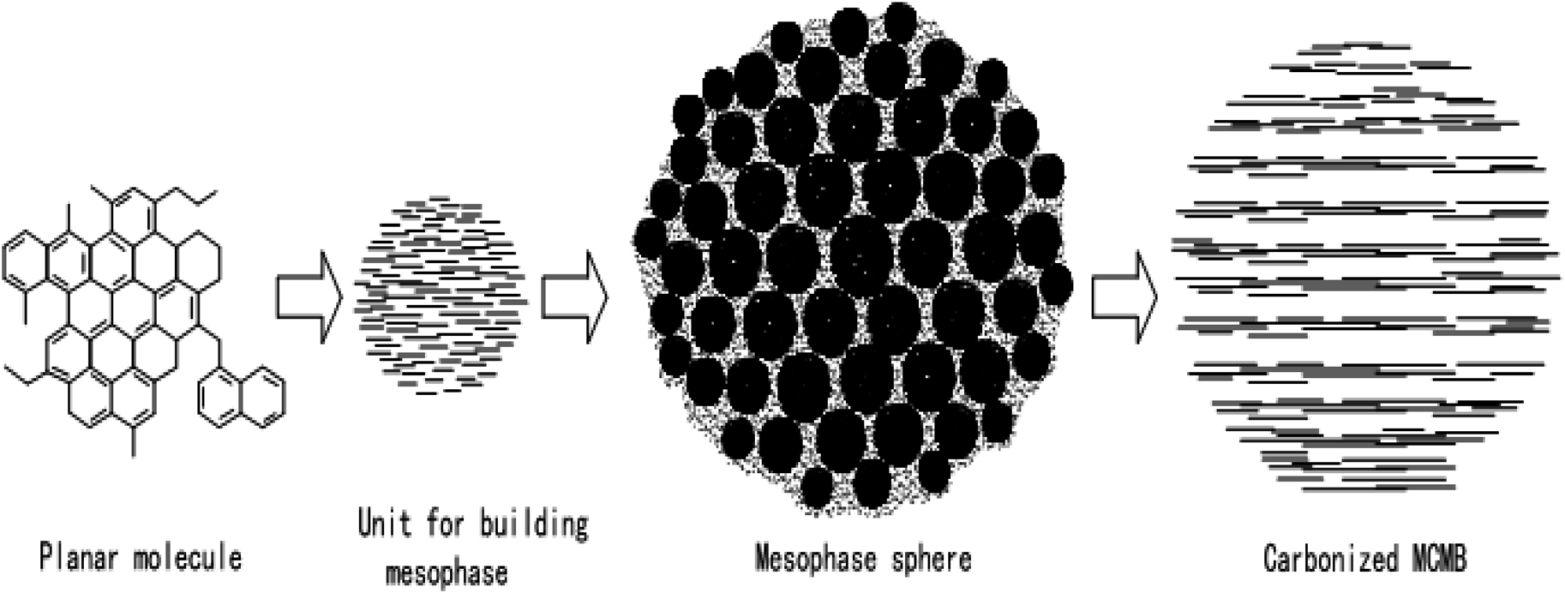
Explanation of the formation process of mesophase in the theory of “Spherical Element Construction”.

Li Tongqi and Wang Chengyang^54,55^ put forward the theory of “Particle Basic Unit Construction” on the basis of in-depth study on the formation process of mesophase carbon microspheres. The theory holds that the formation and development of mesophase serves as a continuous construction process of tertiary structure. First, small aromatic molecules are condensed to form large planar condensed aromatic molecules, which are primary structures. Then these large planar lamellar molecules accumulate to form spherical, rod-shaped or other shaped mesophase building units, which are secondary structures. Finally, these mesophase building units are stacked to form mesophase pellets, which have a three-stage structure. The process is as shown in Fig. 5.

Mesophase spheres grow continuously by the consistent accumulation of building units. When the spheres contact each other, the building units formed will continuously accumulate on the spheres, so that these spheres are connected, and in the meantime, chemical bonding between molecules occurs, which enables the spheres to be merged. After the spheres are merged, their internal structures will be rearranged again, and then they will develop into bulk mesophase. The theory of “Particle Basic Unit Construction” further perfects the theory of “Micro-domain Construction” and acts as the most reasonable one in this field.

### 3.4 Solution theory

In order to accurately and quantitatively study the formation process of mesophase pitch, researchers refer to the Solution theory.^56^ Raw asphalt is a mixture with complex structure, and various kinds of reactions occur during carbonization, therefore it is challenging to quantitatively study the phase equilibrium of mesophase in liquid phase system. At present, there are two methods to quantitatively study the formation of mesophase. One is that Shishio *et al.*^57^ used the liquid crystal theory based on ideal solution to study the phase transformation process of mesophase pitch. He regarded benzene soluble and benzene insoluble components of asphalt as pure components, and assumed that benzene soluble components could not synthesize mesophase, thus forming a binary system. Bates research group^58^ speculated the phase transition of mesophase pitch based on the double potential theory, and concluded that aromatic molecules with high planarization degree are helpful for anisotropic structures to generate nematic liquid crystals.

## 4. Preparation method of mesophase pitch

Researchers at home and abroad have systematically studied the preparation methods of mesophase pitch. The preparation methods can be divided as follows, one-step polycondensation method, two-step polycondensation method, catalytic polycondensation method, pre-mesophase method, potential intermediate phase method and new mesophase method,^59–62^*etc.* And according to the principle and operation technology, the methods can be categorized as follows, direct thermal polycondensation method,^63–65^ co-carbonization method,^66–68^ hydrogen supply modification method,^69–71^ cross-linking modification method^72,73^ and alkylation modification method.^74–76^ The latter will be our next main focus.

### 4.1 Direct thermal polycondensation method

Direct thermal polycondensation serves as the basic method for synthesizing mesophase pitch. The reaction system is overall in a nitrogen environment. After high temperature and high pressure treatment, the raw pitch participates in a series of thermal reactions. During the reaction process, some small gas molecules will escape from the reaction system. Then a series of alignment reactions are generated, resulting in the continuous increase of the degree of molecular condensation, the increase of the viscosity between the reactants, and finally the formation of mesophase pitch. At present, the production rate of anisotropic components in the existing mesophase preparation process reaches up to 100%, and the softening point of the product is about 283 °C.^77^

In 1965, Professor Otani of Gunma University in Japan developed pitch-based carbon fiber for the first time with PVC pitch as raw material. In 1971, Professor Otani summed up his experience after preparing mesophase pitch after trails and errors. Carbon fiber was synthesized from mesophase pitch prepared by direct thermal polycondensation method,^78^ with an average tensile strength of 9.5 × 10^6^ g cm^−2^ and an average modulus of 6.5 × 10^8^ g cm^−2^. If the temperature reaches 2500 °C with appropriate pressure, the tensile strength can reach 1.83 × 10^7^ g cm^−2^ and the modulus 2.1 × 10^9^ g cm^−2^.

Seo and coworkers *etc.*^79^ synthesized a cohesive mesophase pitch through direct thermal polycondensation. The research team found that when the temperature exceeds 400 °C, the small spherical mesophase structure will polymerize into a large mesophase structure. With the increase of heat treatment temperature and the accumulation of reaction time, the softening point, carbon-based ratio and quinoline insoluble content of the product asphalt increase, which further proves the increase of mesophase content. When the second heat treatment is carried out, the hydrogen–carbon ratio of mesophase pitch will be obviously improved, and the aromaticity of mesophase pitch will also be enhanced. Dehydrogenation and polycondensation of aromatic compounds are the main causes for the formation of mesophase.

The researchers represented by Liu^80^ used the direct thermal polycondensation method to analyze the transformation of aromatic hydrocarbons in the initial stage of thermal conversion and its influence on subsequent carbonization behavior. According to their observation, preheating the reaction system at 450–500 °C for a short time will help to transform monocyclic aromatic hydrocarbons and bicyclic aromatic hydrocarbons into polycyclic aromatic hydrocarbons. When the amount of polycyclic aromatic hydrocarbons increases by 14.92%, the synthesis time of mesophase pitch can be shortened by about 2 hours. Moreover, the synthesized mesophase pitch possesses large watershed structure, great lamellar orientation (*d*_002_ = 3.46 Å, *L*_c_ = 21.94 Å), softening point of 273 °C only, therefore the quality can be guaranteed. There are quite a few benefits for the reaction when carrying out thermal pretreatment before direct thermal polycondensation: (1) an increase in the raw materials containing polycyclic aromatic hydrocarbons can accelerate the accumulation of mesophase. (2) The time of mesophase formation can be shortened. (3) The excessive carbonization of molecules with high thermal reactivity is inhibited.

The researchers, in the meantime, observed that the viscosity, flatness, length and content of alkyl side chains of mesophase determine the layered orientation of mesophase products. The addition of aromatic hydrocarbons, meanwhile, can dilute the molecules with high carbonization activity, promote the hydrogen transfer reaction, and act as a stimulant to the formation of planar macromolecules.

The preparation of mesophase pitch by direct thermal polycondensation is relatively simple among all preparation methods. This method, however, has high restrictions on the properties and structure of raw materials, hence only a few raw materials can be employed to prepare mesophase pitch with good performance when adopting this method.

### 4.2 Co-carbonization method

In order to overcome the deficiencies of most raw material properties when preparing mesophase pitch, the co-carbonization method acts as the primary method. Co-carbonization method refers to the addition of co-carbonization agent in the original reaction system, which is to prepare mesophase pitch with good performance by changing the structure composition of the product molecules. In the co-carbonization process, the co-carbonizing agent serves as a reaction crystal nucleus, promoting the system to generate a product with a liquid crystal structure, and at the same time provides hydrogen radicals to the mesophase system, facilitating the hydrogen transfer reaction of the system. At present, there is a production process that can form a 100% streamlined structure mesophase, and the softening point of the product can be controlled between 250 °C and 300 °C by the co-carbonization method.^68,81^

Li and coworkers *etc.*^82^ employed naphthalene pitch as a co-carbonizing agent to co-carbonize with treated FCC slurry to synthesize mesophase pitch. The researchers found that with the increase of the proportion of naphthalene pitch, the solubility of the product in quinoline and toluene and the size of the optical anisotropic component registered an obvious increase, while the softening point decreased, and all aspects of performance improved significantly. The researchers also found that the higher the naphthenic structure in naphthalene pitch, the better the thermal stability of the product. The interaction between naphthenic structure in naphthalene pitch and short-chain alkyl in FCC slurry promoted the increase of molecular weight of the product. Mesophase pitch synthesized by co-carbonization method boasts the advantages of low softening point, high solubility and anisotropic content close to 100%. It is found that the molecular structure of precursor molecules and co-carbonizing agent will directly exert impacts on the experimental effect of co-carbonization. The interaction between the two structures, meanwhile, will affect the yield and stability of mesophase pitch.

Jung and coworkers *etc.*^83^ utilized coal extract as co-carbonization agent to prepare mesophase pitch with petroleum residue. The researchers found that the properties of the product will be obviously improved by adding polyfluoroolefin (PFO) into the system. For example, the lowest softening point is only 230 °C, and when the ratio of coal extract to PFO reaches 2 : 1, the tensile strength of the produced carbon fiber shall reach 1189 MPa. The extract of low rank coal is equipped with high aromatic compounds content, low reactivity, excellent stability and high fixed carbon content. Raw materials, moreover, are affordable, and expensive reaction solvents are not needed, therefore low-rank coal is considered as a co-carbonizing agent with outstanding price-performance ratio.

Fang and coworkers *etc.*^84^ explored the effect of preparing mesophase pitch from waste styrene-butadiene rubber (WSBR) and modified petroleum through co-carbonization process. The results show that when WSBR is employed as modifier, the amount of alkyl groups will rise significantly, which can promote the condensation of aromatic hydrocarbons and is beneficial to the formation of products. When the temperature remains constant, WSBR can enable the product to have better optical anisotropic crystal structure and thermal stability. When the amount of WSBR remained constant, with the increase of temperature, the content of anisotropic structure in mesophase pitch also grew gradually, and the arrangement was increasingly uniform. When the content of WSBR was 10% and the system temperature reached 450 °C, the thermal stability of the product proved to be the best. The researchers also found that the introduction of co-carbonizing agent increased the number of alkyl groups in raw material molecules, and the condensation between aromatic molecules became even more obvious, which promoted the formation of mesophase.

Under the conditions of high temperature and high pressure, the co-carbonizing agent can be utilized as the reaction solvent to reduce the viscosity, improve the environment. And in the end, the reaction process and the performance of the mesophase pitch will get improved. There is a matching effect between the raw material and the co-carbonizing agent, which ultimately affects the modification effect of carbonizing agent on mesophase pitch. There is also a hydrogen transfer reaction in the co-carbonization process, which is beneficial to the thermal reaction. And it also optimizes the raw material pitch in an indirect way, thereby generating a mesophase pitch with excellent performance.

### 4.3 Hydrogen donating modification method

Softening point is an important parameter to evaluate the performance of mesophase pitch. An important indicator that affects the softening point and rheological properties of mesophase pitch is the hydrogen-to-carbon ratio of the raw material. Therefore, hydrogenation modification has a remarkable effect on improving the performance of mesophase pitch. The hydrogen-donating modification method refers to the modification of the mesophase pitch by adding a hydrogen-donating agent to the reaction system, or making the reaction system in a hydrogen environment to control the reaction process. By doing so, the hydrogen–carbon ratio of the product and the properties of mesophase pitches could get improved. At present, there are related processes that can increase the hydrogen–carbon ratio to more than 0.6.^85,86^

Guo and coworkers *etc.*^87^ employed FCC slurry as raw material, and compared the effects of three experimental processes, direct thermal polycondensation, distillation followed by thermal polycondensation and hydrogenation–distillation–thermal poly-condensation, and named the products of these three processes as FCCA, DOA and DOB respectively. The results showed that the ash content of DOB was 25 μg g^−1^, far lower than that of DOA (89 μg g^−1^) and FCCA (1667 μg g^−1^). The content of aromatic hydrocarbons in DOB has been kept at a high level of about 44.45%. The H/C ratio increased from 1.535 to 1.559, and the contents of sulfur and nitrogen in DOB decreased from 0.41% and 0.32% to 0.24% and 0.23% respectively, and the performance of the product appears to be stronger. The researchers also found that in comparison to FCCA and DOA, the content of aliphatic hydrocarbons in DOB showed an apparent increase and the content of alkyl chains was in decline, which indicated that more active hydrogen radicals appeared in DOB during carbonization. Compared with FCCA and DOA, DOB shows a better effect of hydrogen transfer during carbonization, which promotes the fluidity of the reaction system and is easy to form mesophase pitch with large basin structure. Moreover, the content of anisotropic structure and soluble mesophase in DOB system is higher, which shows better flow performance of DOB. At the same time, the lowest softening point of DOB is only 206 °C, and its spinnability is better. The researchers also found that DOB has a better crystal structure and a higher H/C ratio, which proves that the microcrystalline structure of DOB is more orderly than that of DOA and FCCA. To sum up, mesophase pitch with high content, large basin structure, low softening point and orderly microcrystalline structure can be obtained by optimizing the composition and properties of DOB. This acts as a method for converting inferior FCC slurry into high-quality mesophase pitch.

Liu and coworkers *etc.*^71^ added hydrogen donor aromatic oil to the raw asphalt to study the influence of hydrogen donor modification on the product mesophase asphalt. It was found that when the temperature of reaction conditions was 410 °C, reaction time 6 hours, reaction pressure 4 MPa and HAO content 20%, the best reaction effect can materialize. At the same time, the researchers found that the hydrogen transfer reaction and dilution effect were obviously improved after adding hydrogen donor into the system, which was beneficial to improve the mutual compatibility between solvent subcomponents, and then promoted the formation of mesophase. It was also found that mesophase products with high slag rate and excellent anisotropic structure can be obtained by controlling oxidation degree and co-carbonization additives and combining hydrogen modification with subsequent co-carbonization process.

Yu and coworkers *etc.*^88^ employed tetralin as hydrogen donor to investigate the effect of process hydrogenation on mesophase pitch. The researchers divided the experiment into three control groups, group A, group B and group C, and each control group included two heating stages, in which no hydrogen donor was added to the two stages in group A, and the product was named N_1_. In group B, hydrogen donor was added only in the first stage, and the product was N_2_. In group C, hydrogen donors were added in both heating stages, and the product was named N_3_. It is found that the average softening point of N_1_ is 297 °C, N_2_ 253 °C and N_3_ 239 °C. The product's optical watershed structure area follows such order, N_3_ first, followed by N_2_ and N_1_. Raman spectra also shows that the graphitization degree of N_2_ and N_3_ is better than that of N_1_, since N_2_ and N_3_ contain rich naphthenic structures. XRD characterization also demonstrates that the crystal structure among N_1_, N_2_ and N_3_ is improving. At the same time, the researchers found that the process hydrogenation method is beneficial to the formation of hydrogenation intermediates with uniform molecular structure and rich cycloalkyl structure. In addition, adding methylene bridge to the product also plays an important role in improving the performance of the product, which is similar to the research result of Guo research group.^87^

Currently, there are two types of hydrogen-donating modification methods that have been industrialized, namely, indirect hydrogenation and direct hydrogenation. After the reaction system is modified by hydrogen-donation, the naphthenic structure and the hydrogen–carbon ratio are on the rise, resulting in a decrease in the viscosity of the reaction system. Based on this, the formation of mesophase pitch can be promoted.

### 4.4 Cross-linking modification method

The specific operation of the cross-linking modification method is to add a cross-linking agent to the raw asphalt, so that the asphalt forms macromolecules under the action of the cross-linking agent. The performance of the mesophase asphalt, finally, will be enhanced. Whether it can provide an unsaturation is the main indicator for selecting a crosslinking agent.

Kim and coworkers *etc.*^89^ introduced nitrogen and oxygen functional groups on the molecular surface of raw asphalt, and increased the molecular weight of asphalt through cross-linking modification. The researchers employed pitch-based activated carbon as raw material, modified it with nitric acid, and treated it with blank control. The results show that the specific surface area of the sample without nitric acid modification is only 16.3 m^2^ g^−1^, while the specific surface area of the sample with nitric acid modification can reach 39.5 m^2^ g^−1^. And with the increase of nitric acid concentration, the specific surface area gradually increases, and the highest specific surface area can reach 967.7 m^2^ g^−1^. When the mass fraction of nitric acid is 10%, the activity of asphalt is the highest. During the research, it was found that the introduced functional groups were removed during carbonization, so it exerted no direct influence on the physical activation process of the experiment. With the rise of molecular weight of mesophase pitch, the softening point also increased, which hindered the rearrangement of pitch molecules during carbonization, thus inhibiting the optimization of pitch molecular orientation and reducing the crystallinity of mesophase pitch. The decrease of crystallinity increases the reactivity of carbonized asphalt with activator, forming a microporous structure, and then preparing activated carbon with high specific surface area.

Zhang and coworkers *etc.*^90^ prepared mesophase pitch with sulfur as crosslinking agent, furfural essential oil as plasticizer and sulfur as crosslinking agent. The researchers found that when the mass fraction of SBS was 6%, plasticizer was 4% and crosslinking agent was 0.2%, the best experimental effect could be reached. Crosslinking agent improves the anti-aging performance of products and the compatibility between asphalt and SBS. The participation of plasticizer promotes the swelling and dispersion of SBS in asphalt, further improves the effect of crosslinking agent, reduces the rutting resistance of asphalt after aging, and improves the viscosity of asphalt. The effect of crosslinking agent depends on the swelling degree of SBS to some extent. Due to the cross-linking reaction, a cross-linked polymer network was formed in mesophase pitch, which greatly improved the stability of the product. At the same time, the researchers also found that the combination of plasticizer and cross-linking agent can play a better role. Plasticizer can improve the compression resistance of mesophase pitch, and can also promote SBS to expand further, which is beneficial to the cross-linking agent.

It can be observed, based on above-mentioned findings, that if the temperature in the system gets too high, the polymerization reaction of the crosslinking agent and the raw materials will be rather difficult to control. Since almost all the process routes for preparing mesophase pitch are carried out under high temperature conditions, therefore the crosslinking modification method is rarely used to prepare mesophase asphalt at current stage.

### 4.5 Alkylation modification method

Alkylation modification method also acts as a very popular method for the preparation of mesophase asphalt. Alkylation modification refers to the introduction of alkyl groups into the molecules of the raw asphalt to increase the alkane structure in the product. This helps to reduce the viscosity of the mesophase asphalt, and then affects the lamellar structure arrangement between the product molecules, and finally improves the properties of asphalt. At present, there is an alkylation modification process that can increase the anisotropic content of mesophase pitch by more than 70%, and a product with a tensile strength of 1190 MPa can be obtained by the alkylation modification method.^83,91^

Liu and coworkers *etc.*^92^ employed tetrahydronaphthalene and polyethylene glycol as modified materials to prepare mesophase pitch by direct polycondensation, and explored the influence of naphthenic structure and alkyl chain in modified materials on the formation and development of mesophase. The specific operations are as follows: the raw materials without modifier are used as the blank control group, and the experimental groups with 5%, 10%, 20% and 30% tetralin respectively, and the experimental groups with 5%, 10%, 20% and 30% polyethylene glycol are introduced at the same time. The results show that with the increase of tetralin content, the hydrogen–carbon ratio of the products gradually increases, reaching the highest of 1.1927, the lowest softening point of 218 °C, and the carbon residue of 85.57%, and the products are all large basin mesophase pitch, and therefore great experimental effect could be expected. When the content of polyethylene glycol increases, the hydrogen–carbon ratio first increases and then decreases, and the highest is 1.1616, the lowest softening point is 226 °C, and the highest carbon residue occupies 83.76%. Some products are mesophase pitch. However, in the blank control group, the hydrogen–carbon ratio is only 1.1180, the softening point is 259 °C, and the carbon residue takes up only 73.68%. There is almost no mesophase pitch with large basin structure in the product, and its performance is much worse than that of the experimental group with alkylation modifier. It can be concluded that the existence of alkyl structure in modified materials is conducive to the formation of high-quality mesophase, in which naphthenic structure is better than alkyl chain. The researchers also found that if the raw materials contain rich alkyl structure, it is helpful to prepare high-quality asphalt with wide-area structure, low softening point, high carbon residue value and ordered microcrystalline structure, which is similar to the research results of Guo team.^15^

Wu and coworkers *etc.*^85^ prepared mesophase pitch from coal tar pitch (CLP) with tetralin as alkylation modifier and explored the best reaction conditions. The results showed that when the addition of tetralin was 12.5% and the temperature reached 400 °C, the hydrogen–carbon ratio of the product could reach 0.64, and the content of the generated mesophase could reach 76%, which was 33.5% higher than that of the component without alkylation modifier. The researchers found that with the increase of alkylation modifier, the content of cycloalkyl and methylene structure showed an upward trend. Appropriate naphthenic side chains and alkane structures are beneficial to the formation of fiber-like and flake-like optical textures, while long aliphatic side chains are conductive to the formation of mesophase mosaic optical textures.

Wang and coworkers *etc.*^93^ employed biomass pitch (BTP) as modifier to explore the influence of modifier on the preparation of mesophase pitch. The results showed that with the increase of BTP, the softening point of the product decreased continuously, and the lowest was only 301.6 °C. Impurities such as quinoline insolubles, sulfur compounds and nitrogen compounds gradually decreased, and they could decrease at least below 0.1%. The content of the product also increased, with a gradual rise up to the highest of 0.581 in the hydrogen–carbon ratio. The optical watershed structure of the product also appeared to be larger with the increase of BTP. At the same time, it was found that after adding biomass pitch, the number of alkyl and cycloalkyl groups in the reaction system was on the rise, and the viscosity, softening point and wide-area structure of mesophase pitch decreased, which was more conducive to the development of mesophase and high uniaxial arrangement. The increase in the number of alkyl and cycloalkyl groups greatly improved the performance and yield of the product.

Based on these findings, it can be inferred that the performance of mesophase pitch is directly affected by the alkyl structure in the raw material pitch. Since it is too difficult, however, to introduce the alkyl structure into the raw material molecules and there is no suitable industrial catalyst to participate in the reaction, therefore it is only at the initial stage.

## 5. Impact factors for the formation of mesophase pitch

The effect of FCC slurry on the synthesis of mesophase pitch is not only affected by the preparation process, but also by some basic factors on the formation of the products. The effects of raw materials,^94–96^ temperature,^97–99^ pressure,^100,101^ reaction time,^80,102^ system environment^103,104^ and stirring rate^105–107^ on the formation of mesophase pitch in the reaction system are reviewed below.

### 5.1 Impact of raw materials

**Fig. 6:**
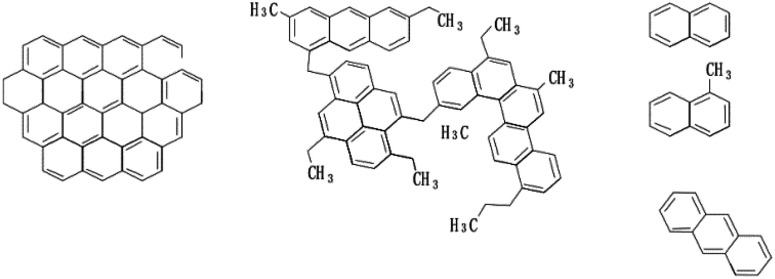
Molecular structure of coal tar pitch, petroleum pitch and naphthalene compounds (from left to right).

The reference indicators of high-quality mesophase pitch are softening point and rheological property. The molecular structure and properties of raw materials can directly affect these two parameters. For the moment, the main preparation raw materials of mesophase pitch in the chemical industry include aromatic compounds, petroleum pitch, naphthalene pitch and coal tar pitch,^108^*etc.*Fig. 6 shows the molecular structure model of three commonly used raw materials.

According to a large number of research data, it is concluded that coal tar pitch is the most frequently used raw material in that it is rich in sources and easy to obtain; however, most of the mesophase pitch prepared from coal pitch has unsatisfactory yields and activities.^17,109^ Petroleum pitch contains relatively more alkyl, so the mesophase pitch prepared has better activity and yield.^84,110^ When using aromatics as raw materials to prepare mesophase pitch, the purity of aromatics is required to be extremely high, and the reaction conditions are severe, which makes the quality of mesophase pitch relatively the best.^111–113^

At present, aromatic components act as the preferred raw materials for the preparation of high-quality mesophase pitch mainly for their uniform molecular composition, appropriate hydrogen–carbon ratio and low impurity content. In addition, the activity and yield of mesophase pitch can be regulated by controlling the content of alkanes on the molecular side chains of raw materials.

### 5.2 Impact of reaction temperature

It is very vital to control the reaction temperature during the whole reaction as the whole reaction process of synthesizing mesophase pitch belongs to free radical reaction.

When the reaction system starts to heat up, the reaction rate will increase significantly, which is conducive to the condensation polymerization and thermal cracking of raw pitch, and also shortens the time for the formation of mesophase pitch. When the temperature exceeds the critical value, however, the reaction system is inclined to be out of control, with excessive condensation of raw pitch molecules and eventual carbonization of products.^17,114^

According to the current research data, the optimal temperature for the formation of mesophase pitch is about 400 °C. The performance of mesophase pitch will be seriously affected by either too high or too low temperature in the reaction system while the main indicators for evaluating its performance are the size of the product molecules and their degree of polymerization.

### 5.3 Impact of reaction pressure

In addition to raw materials and reaction temperature, reaction pressure also affects the formation of mesophase pitch. During the formation of mesophase pitch, proper separation of light components can reduce the viscosity of raw pitch, increase the chance of inter-molecular collision, and then promote the synthesis of mesophase pitch.

During the reaction process, the system pressure could be adjusted duly to control the viscosity of the raw materials in the reaction system, contributing to the orderly accumulation of planar aromatic hydrocarbon molecules and the fusion and growth of mesophase spherule. Reasonable adjustment of the system pressure can optimize the product composition and reduce the softening point and porosity of the product. And in the meantime, the yield of solid products could be improved so as to obtain mesophase pitch with excellent performance.^114,115^

When the pressure in the reaction system gets too low, the small molecules of gas generated from the thermal cracking of the raw pitch will escape from the reaction system with the light components in the system, which greatly reduces the yield of mesophase pitch, and also significantly reducing the content of alkyl structure in the reaction system, resulting in a significant increase in the softening point of mesophase pitch, which seriously affects its performance. When the pressure in the system gets too high, there remains difficulty for the light components in the system to escape, which damages the anisotropy of the product molecules, leading to a significant decline in the performance of mesophase pitch. Therefore, mastering the method to properly control the system pressure becomes an important task for synthesizing high-quality mesophase pitch.

### 5.4 Impact of reaction time

In the process of preparing mesophase pitch, controlling the reaction time has a significant effect on the properties, optical structure and final molecular structure of the product.

Under the suitable temperature and pressure conditions, the thermal cracking reaction continues to occur in the reaction system with the increased reaction time. Some small alkane molecules accompanied by the escape of light components are then generated. Meanwhile, condensation polymerization occurs between the aromatic groups in the raw material to form macromolecules which will continue to participate in the aromatization and thick cyclization reaction to produce intermediate product, planar polycyclic aromatic macromolecules. And the intermediate product will further form spherical mesophase under the external force. The mesophase spherule will continue to fuse, grow and then break into the mesophase pitch when it reaches the critical value.^116,117^

If the reaction time lasts too long, the raw pitch will overreact, with the product being over condensed. Then it might be rather hard for the product molecules to accumulate orderly due to their oversized condition, leading to a significant decline in product performance. In contrast, if the reaction time lasts too short, the mesophase spherule is not fully developed. Then it will be quite challenging to fuse or grow, resulting in the poor performance of the synthesized mesophase pitch with a mosaic optical structure. Therefore, controlling the reaction time plays a significant role in the ultimate product properties.

### 5.5 Effect of reaction atmosphere

In the process of mesophase pitch preparation, in order to ensure the pressure stability of the experimental system and inhibit the oxidation of the reactants in the system, researchers mostly fill the reaction system with some protective gases to ensure the stability of the whole reaction. The protective gases, most commonly used in the current mesophase pitch preparation process, are nitrogen and argon gas.^118,119^

Klaus J. and coworkers *etc.*^120^ adopted CO_2_ as the shielding gas to explore its influence on the formation process of mesophase pitch. The researchers found that CO_2_ can accelerate the formation of polyaromatic hydrocarbons as well as the formation of mesophase. The researchers also found that the glass transition temperature of the mesophase product mainly depends on the content of the mesophase or insoluble substance, which bears little relationship with the type of shielding gas in the reaction system.

Klaus J. and coworkers *etc.*^121^ found that hydrogen can also be used as protective gas, and the mesophase pitch synthesized in hydrogen atmosphere has better performance and higher reactivity compared to that in nitrogen or argon atmosphere. According to the researchers, the activation energy of reactants in hydrogen atmosphere is also lower, which allows for faster and more energy efficient reaction conditions. Moreover, the researchers observed that the products had higher glass transition temperature and wider glass transition range, which indicates that the raw pitch had higher average molecular weight with wider distribution in the hydrogen atmosphere.

Based on the above contents combined with a large number of previous research data, we can draw a conclusion that nitrogen as a protective gas may have an inhibitory effect on the mesophase pitch while argon or carbon dioxide as a protective gas can promote its formation. Hydrogen is the best choice among the known shielding gases, as in line with 3.3 above,^71,85–88^ hydrogen can not only be used as a protective gas, but also can react with the raw pitch molecules for hydrogenation. The hydrogen carbon value of mesophase pitch can get increased, its softening point reduced, its optical structure optimized. And then mesophase pitch with better quality can be prepared.

### 5.6 Impact of mixing rate

In addition to the above factors, there is another easily neglected but equally important factor: the mixing rate which will ultimately affect the performance of the product by participating in the formation process of mesophase pitch.

The mixing operation can provide a buffering effect for the reactants during the reaction to avoid excessive growth of the mesophase spherule, which in turn can adjust the structure and appearance of the product molecules, thus improving the properties of the mesophase pitch.^122^

During the preparation of mesophase pitch, the raw pitch is mixed evenly through stirring to ensure the homogeneity of the subsequent reaction. At the same time, stirring can accelerate the mass and heat transfer in the reaction system, which is conducive to maintaining the stability of the reaction.

## 6. Application of mesophase pitch

Mesophase pitch has become the first choice for high-quality carbon material precursors for its numeral excellent properties, receiving much attention in the fields of high-tech materials such as refractories,^123–127^ energy storage materials, and electrode materials. The post-treatment conditions and structure of mesophase pitch determine the performance and structure of the prepared carbon materials which include carbon fibers, needle coke, porous carbon, carbon microbeads, and foam carbon.^128–131^

### 6.1 Mesophase pitch-based carbon fiber

In addition to its low density, high modulus, high strength, carbon fiber also has the resistance to deformation and high temperature, small coefficient of thermal expansion, good mechanical properties as well as thermal and electrical conductivity. Carbon fiber materials can be used as both structural and functional materials. High-performance carbon fiber bears high specific modulus and strength. The density of high-quality carbon fiber is only about 25% of that of steel wire while the tensile strength can reach 3 times that of steel wire, and the tensile modulus can even reach 5 times that of steel wire, which is the best material among high-performance fiber materials. Carbon fiber and its composites are widely used in aviation, industrial and medical fields.^132,133^

### 6.2 Needle coke

In terms of raw materials for needle coke preparation, needle coke can be divided into oil-based needle coke and coal-based needle coke. The main raw materials for coal-based needle coke include coal tar and coal tar pitch while the main raw materials for oil-based needle coke include thermal cracking residue, ethylene cracking residue, *etc.* The preparation process of needle coke includes three stages: raw material pre-treatment, delayed coking and calcination. In addition to its high degree of graphitization and satisfactory electrical conductivity, needle coke has relatively large lithium storage capacity and higher theoretical capacity and better low-temperature performance than graphite-based materials. Electrode steel making of needle coke saves 30% to 50% time and consumes 20% to 50% less energy than ordinary electric grade. Needle coke materials have been widely used in electro-chemical capacitors, graphene preparation and other fields at present.^18,34^

### 6.3 Mesophase pitch-based foam carbon

Mesophase pitch-based foam carbon works as a lightweight porous material with a three-dimensional network structure composed of pores and pore wall groups connected to each other, which not only has conventional carbon material properties, but also has high electrical conductivity, high thermal conductivity, low expansion coefficient and corrosion resistance. The main raw materials of mesophase pitch-based foam carbon include coal pitch, petroleum pitch and naphthalene pitch. The raw materials are treated to obtain mesophase pitch, and then carbonized, foamed and graphitized to finally obtain pitch-based foam carbon. After graphitization, the mesophase pitch-based foam carbon takes on a new type of graphite ligament structure, which greatly improves the original mechanical and thermal properties. The performance of foam carbon can be improved by changing the process parameters (such as foaming pressure, heating rate, *etc.*). The researchers also found that the foam carbon with better performance can be obtained by extracting and pre-treating mesophase pitch and the pitch-based foam carbon is widely used in electrode materials, electromagnetic shielding materials, adsorption materials and other civilian fields.^132,134–136^

### 6.4 Mesocarbon microbeads

Mesocarbon microbeads are a brand-new type of carbon material whose development originated from the process of mesophase formation, and whose main raw materials include petroleum pitch, pure aromatics or coal tar pitch. They are of great interest to researchers because of their advantages of high density, high purity and high strength. The preparation methods of mesocarbon microbeads include suspension method, emulsification method and poly-condensation method. The process core of each preparation method is the formation, separation and carbonization of mesophase spherule. After the mesocarbon microbeads are formed, they will be free in the mother liquor. Besides, the separation method is vital in order to obtain high performance mesocarbon microbeads. The currently known separation methods include centrifugal separation, thermal filtration and solvent separation methods whose ultimate products are widely used in chemical, nuclear, semiconductor, new energy vehicles and other industries.^10–12,137^

### 6.5 Porous carbon

Porous carbon also serves as a new type of carbon material. The raw materials for preparing porous carbon include mineral materials and biomass materials, among which petroleum pitch is one of the most suitable raw materials. Porous carbon materials have the advantages of uniform ion and electron diffusion paths, *etc.*, which have attracted the attention of researchers. The commonly used preparation methods of porous carbon materials include template method, carbonization method, activation method and catalytic method. Most of the hierarchical porous carbon materials have multi-level pore structures, which can be fully contacted with ions and molecules both internally and externally, so the electrode materials usually have the advantages of short diffusion distance, high efficiency of current transmission as well as many effective storage sites. Porous carbon materials are widely used in the fields of energy storage, energy conversion,^132,138^*etc.*

The above-mentioned advanced carbon materials prepared with mesophase pitch as precursors have the common features of affordable cost, stable structure and abundant sources, *etc.* In addition to their high fiber content, most of the carbon materials prepared from them have corrosion resistance, impact resistance, high strength, high temperature resistance, high carbon content, high density, satisfactory thermal and electrical conductivity and stable chemical properties. Compared with traditional materials, advanced carbon materials are cheaper, moreover, have better performance than traditional materials. Therefore, they are recommended for wide promotion and application in various fields.

## 7. Overview and outlook

The investigation results in recent years show that the proportion of mesophase pitch made from petroleum pitch is getting higher and higher due to its affordable cost, wide source and high aromatic structure content. FCC slurry has also become a common raw material for preparing mesophase pitch, and the performance of mesophase pitch can be adjusted by selecting different preparation processes. The direct thermal condensation polymerization method can be adopted as a easily conducted one. This method, however, has strict requirements on the structure and properties of raw materials, and many raw materials are not suitable because of their poor quality. If the quality of raw materials are not that favorable, the mesophase pitch can be prepared by co-carbonization or cross-linking modification method. However, the application of cross linking modification method is based on the premise that there must be a cross-linking agent that provides unsaturation, and it is rather difficult to control the degree of polymerization under high temperature conditions. The co-carbonization method is employed to change the reaction system by adding co-carbonization agents, thus changing the molecular structure of the product in order to obtain mesophase pitch with excellent properties. If the softening point is required to be relatively high, the hydrogen supply modification method can be used to directly increase the hydrogen-to-carbon ratio of the product, thus improving the performance of the product. Researchers have found that the introduction of alkanes into the raw pitch can improve the performance and optical structure of the mesophase pitch, where the introduction of cycloalkanes is more effective than the introduction of chain hydrocarbons. Therefore, it is possible to introduce alkyl structures into the raw pitch molecules through the alkylation modification method. The operation process of this method is, however, relatively complex, and the catalyst technology needs to be improved, therefore there is no large-scale industrialization yet. The process of preparing mesophase pitch is not only related to the preparation method, some basic factors can also affect the formation of mesophase pitch, such as raw materials, temperature, pressure, time, gas environment and mixing rate. Controlling these conditions, therefore, can also be quite helpful in the formation of mesophase pitch. The pre-oxidation process of mesophase asphalt can convert methyl and ethyl groups on asphalt molecules into carbonyl and carboxyl groups, improve the melting point of the products, and prevent the products from melting or melting in the subsequent carbonization and graphitization. Compared with traditional materials, the advanced carbon materials prepared by mesophase pitch have more stable structure and better performance. And with lower cost, also they are more friendly to the environment, therefore they have already been widely promoted and applied across fields.

Despite quite a few satisfactory results achieved by FCC slurry in the study of preparing mesophase pitch, there are still much space to be improved. Some suggestions, therefore, are presented as below,^139–141^

(1) The research on the technology and reaction mechanism of co-carbonization, the pilot research on co-carbonization, and systematic research for large-scale industrial application can be carried out.

(2) In view of the fact that the performance of mesophase pitch prepared by co-carbonization method mainly depends on the characteristics of raw materials, the directional pretreatment of key components and heavy aromatics in mesophase pitch prepared by co-carbonization method should be strengthened.

(3) Attention should be paid to the research on structure–activity relationship, phase formation mechanism and molecular structure of mesophase pitch, and by exploring the relationship between molecular structure of mesophase pitch and phase formation behavior and spinnability of pitch, the goal of improving spinnability and the preparation quality of carbon fiber can be achieved.

(4) The cooperation of related enterprises, R&D and production of mesophase pitch should be strengthened, so that high-quality raw materials for preparing mesophase pitch that is used to produce carbon fibers can be provided.

## Conflicts of interest

Authors declare no competing interest.

## Supplementary Material
